# Haemophagocytic lymphohistiocitosis - a case report in infant

**DOI:** 10.1186/1939-4551-8-S1-A76

**Published:** 2015-04-08

**Authors:** Eliana Toledo

**Affiliations:** 1 Faculty of Medicine of São José Do Rio Preto, Brazil

## Background

The purpose of this case report is to alert for a serious disease, potentially lethal, often confused with sepsis, however, it is an auto inflammatory disease, with massive activation of macrophages and consequent tissue destruction.

## Methods

Review of electronic medical record.

## Results

We report a case of an infant, 1 year and 8 months old, male, that was in intensive care unit for five months with initial diagnosis of acute diarrhea, dehydration and hemolytic uremic syndrome.

The infant developed fever, hepatosplenomegaly, cutaneous rash, bicytopenia, elevated serum ferritin, liver enzyme abnormalities, encephalitis, acute renal failure and serum hypogammaglobulinemia.

The diagnosis of hemophagocytic lymphohistiocytosis was suspected and confirmed with biopsy of bone marrow with evidence of hemophagocytosis. Chemotherapy was instituted promptly with etoposide, dexamethasone and intravenous human immunoglobulin (immunomodulation dosis), with a favorable evolution and discharge from the intensive care unit.

## Conclusions

We call attention to the clinical and laboratory diagnosis of hemophagocytic histiocytosis which is a potentially lethal disease if not diagnosed early.

## Consent

Written informed consent was obtained from the patient for publication of this abstract and any accompanying images. A copy of the written consent is available for review by the Editor of this journal.

